# The Daily Association Between Leisure Activity Engagement and Cognitive Function

**DOI:** 10.1093/geroni/igaf122.1179

**Published:** 2025-12-31

**Authors:** Angie Sardina, Alexa Allan, Lesley Ross, Alyssa Gamaldo

**Affiliations:** Clemson University, Clemson, South Carolina, United States; The Pennsylvania State University, University Park, Pennsylvania, United States; Clemson University, Clemson, South Carolina, United States; Clemson University, Clemson, South Carolina, United States

## Abstract

Regular leisure activity engagement (LAE) is a protective factor for maintaining cognition and reducing Alzheimer’s disease and related dementia (ADRD) risk. However, limited research has explored the association between daily LAE and cognition despite evidence of both LAE and cognition varying daily in sociodemographic heterogenous aging populations. Thus, this study examined 1) intraindividual variability in daily LAE; 2) the association between LAE and cognitive functioning (within-person and between-person); and 3) whether the LAE and cognitive association varies by sociodemographic characteristics. Data from the Healthy Aging in Neighborhoods of Diversity Across the Life Span (HANDLS) Sleep sub-study were utilized. Using a mobile device, participants (N = 211; age range=46-83; 68% Black; 73% female) reported their daily participation in 56 leisure activities (e.g., choir, reading, jogging, watching tv) across a 7-day period. Participants also completed brief cognitive assessments of memory (DOT memory) and processing speed (Symbol Search). Participants, on average, engaged in seven leisure activities/day (SD = 3.5). Multi-level models revealed significant intraindividual variability observed in LAE with 50% of LAE observed at both within- and between-person levels. Individuals who reported more daily LAE tended to perform significantly better on the memory task [b=-0.04, SE = 0.02, p = 0.04]. No significant within-person associations between LAE and cognition were observed. No significant interactions between LAE and sociodemographic characteristics were observed for either cognitive measure. These findings emphasize the importance of routine, daily, LAE on everyday cognition, which may elicit longer-term protective effects against cognitive decline and ADRD. Future research should explore psychosocial and contextual factors that potentially moderate these relationships.

